# German Delphi consensus: COMT inhibitors in the management of wearing-off fluctuations in Parkinson’s disease

**DOI:** 10.1007/s00702-026-03278-x

**Published:** 2026-09-12

**Authors:** W. H. Jost, R. Müller, R. Schüle, A. Storch, K. Witt, L. Tönges

**Affiliations:** 1 Parkinson Klinik Ortenau, Kreuzbergstraße 12-16, 77709 Wolfach, Germany; 2 Department of Neurology, Saarland University, Homburg, Germany; 3 Department of Neurology, University Hospital Augsburg, Augsburg, Germany; 4 Department of Neurodegenerative Diseases and Movement Disorders, Department of Neurology, Faculty of Medicine, Heidelberg University Hospital, Heidelberg, Germany; 5 Centre for Neurology and Hertie Institute for Clinical Brain Research, University of Tübingen, Tübingen, Germany; 6 Department of Neurology, University of RostockGerman Centre for Neurodegenerative Diseases (DZNE) Rostock-Greifswald, Rostock, Germany; 7 Department of Neurology, Evangelical Hospital Oldenburg, and Research Centre for Neurosensory Sciences, Carl von Ossietzky University, Oldenburg, Germany; 8 Tönges, Niermann & Baumgärtner Group Practice, Wuppertal, Germany; 9 Department of Neurology, Ruhr University Bochum, St Josef Hospital Bochum, Bochum, Germany

**Keywords:** Parkinson’s disease, COMT inhibitors, Motor fluctuations, Delphi survey

## Abstract

COMT inhibitors (COMT-Is) have now been established for thirty years as adjunctive medication in the treatment of Parkinson’s disease (PD). When they were first introduced, the prolonged duration of action of levodopa and the increase in levodopa levels were regarded as significant advances. Many questions regarding their practical application have not been fully addressed scientifically. This has led to a degree of uncertainty regarding the assessment of the efficacy and, consequently, the use of COMT-Is. For this reason, the aim of this Delphi consensus was to assess the current status of COMT inhibition in PD in Germany, to identify any shortcomings, and to find new approaches to close our knowledge gaps and reach a consensus on recommendations. In doing so, the authors who formulated the questions for the Delphi consensus aim to provide their colleagues with practical guidance on the use of COMT-Is in the treatment of Parkinson’s symptoms. To achieve this aim, 200 specialists in movement disorders across Germany were asked to evaluate 22 statements across three subject areas. 115 specialists responded. Key findings: In topic area 1, ‘Response fluctuations and the use of COMT-Is’, it emerged that the Wearing-off Questionnaire, 9-item version (WOQ-9) was not unanimously regarded as a suitable tool for diagnosing response fluctuations (agreement rate: 64%). In topic area 2 (role of COMT-Is in the treatment of fluctuations in efficacy), there was a contradiction in that while COMT-Is were not considered the first-line treatment of choice (agreement rate 58%), respondents agreed that they should be used at an early stage when fluctuations in efficacy occur (agreement rate 95%). In topic area 3 (comparison of various aspects of individual COMT-Is), opicapone was unanimously rated as the easiest to manage in daily use due to its once-daily dosing and favourable safety profile (agreement rate: 83%). In summary, the survey provides significant insights into fluctuations diagnosis and the current role of COMT-I in the treatment of PD. The efficacy and tolerability/safety of opicapone observed in clinical trials for the treatment of Parkinson’s patients with motor fluctuations are also confirmed in this survey in clinical practice.

## Introduction

Over the past five decades, various classes of substances and numerous drugs have been introduced into the pharmacological treatment of Parkinson’s disease (PD), most of which directly or indirectly improve dopaminergic stimulation (Höglinger et al. 2023). Levodopa remains the gold standard of pharmacological therapy (Oertel et al. 2011). To prolong its half-life, levodopa is administered in combination with a dopa decarboxylase inhibitor (DDC-I), which inhibits the peripheral breakdown of levodopa via dopa decarboxylase (Tambasco et al. 2018). 

Despite the addition of a DDC inhibitor, the short half-life of levodopa, combined with the progressive pathological loss of dopaminergic neurons in the striatum, leads to wearing-off fluctuations (‘OFF’ phases) as the condition progresses (Tambasco et al. 2018, LeWitt 2015). The management of these motor fluctuations can pose a significant challenge for patients and their treating physicians even in the early stages of PD.

There are a number of strategies for managing wearing-off fluctuations. These include the inhibition of catechol-O-methyltransferase (COMT) (Tambasco et al. 2018). COMT inhibitors (COMT-Is) have been available for around 30 years as an add-on to levodopa/DDC-I therapy. They improve the availability of levodopa, as in the presence of a DDC-I, COMT – which catalyzes the conversion of levodopa to 3-O-methyldopa (3-OMD) in the brain and periphery – becomes the most important metabolizing enzyme for levodopa (Tambasco et al. 2018). 

The first approved COMT-I was tolcapone, for which there is very good efficacy data (Regensburger et al. 2023). As the first approved COMT-I inhibitor, it not only inhibits COMT in the periphery but is also believed to have a central effect on COMT. Due to its relatively short half-life, tolcapone must be taken three times daily (Product 2022). Treatment with tolcapone is limited by side effects, as liver damage and severe diarrhea have been observed during treatment (Regensburger et al. 2023). For this reason, tolcapone was only granted EU marketing authorization for the treatment of patients in whom other COMT-I are insufficiently effective or well-tolerated (Regensburger et al. 2023). The current German S2K guideline lists tolcapone as a second-line treatment (Höglinger et al. 2023).

Entacapone is the second approved COMT-I (Regensburger et al. 2023). It is approved for use in combination with standard levodopa/DDC-I preparations in adult patients with PD who experience ‘end-of-dose’ fluctuations and whose condition is not adequately stabilized by these combination preparations (Product 2024). Entacapone also has a relatively short half-life and must therefore be taken several times a day. Severe diarrhea has been observed in some patients whilst taking the drug. Furthermore, the metabolism of entacapone produces a highly colored breakdown product (Product 2024). Many patients and carers find the resulting discoloration of urine unpleasant and disturbing.

Since 2016, opicapone, a third-generation COMT-I, has been approved as an adjunctive therapy to levodopa/DDC-I in adult patients with PD and motor ‘end-of-dose’ fluctuations, in whom no stabilization can be achieved with these combinations (Regensburger et al. 2023, Product 2022). Opicapone is taken just once a day, is at least as efficacious as other COMT-I, shows no liver toxicity and does not discolor the urine (Observations made by the authors in a very large number of patients).

## Study objective

Using a survey based on the Delphi method, the authors sought to determine the current status of COMT-I use in Germany. The aim was to identify potential shortcomings and to find new approaches for closing knowledge gaps and reaching a consensus on joint recommendations. The findings are intended to provide colleagues with practical guidance on the use of COMT-Is in the treatment of PD.

## Method

### The Delphi method (Antonini et al. 2023)

The Delphi method is an established procedure for consensus-building, in which the degree of agreement is recorded anonymously using a Likert scale from 1 to 5 (1 = complete disagreement, 5 = complete agreement). If more than 75% of participants selected 4 or 5 as their response, this was counted as an affirmative response. The corresponding percentage is shown in Table 1 and below, next to each statement. The authors further specified that if between 75% and 10% of participants selected 4 or 5, this response was classified as ambiguous and if fewer than 10% selected 4 or 5 as their response, the response was classified as rejection.

**Table 1:** Survey statements and proportion of respondents who agreed or strongly agreed with the statement (4 or 5 on the Likert scale); Evaluation: >75% = affirmative response (white), < 75% to 10%: ambiguous response (light grey), < 10%: negative response (dark grey) Topic 1: Fluctuations in efficacy and the use of COMT-Is

*Topic 1: Fluctuations in efficacy and the use of COMT-Is*		
1.1	Variations in effect can manifest themselves in both motor and non-motor behaviour.	99.1%
1.2	Motor fluctuations are diagnosed on the basis of the patient’s medical history and clinical examination.	93%
1.3	Non-motor fluctuations are diagnosed on the basis of the patient’s medical history and clinical/neuropsychiatric examination.	89.6%
1.4	The assessment of motor and non-motor fluctuations requires systematic evaluations, such as the WOQ-9.	64%
1.5	COMT-Is are suitable for the treatment of motor and non-motor fluctuations in Parkinson’s patients.	97.4%
1.6	COMT-Is may also be suitable for the treatment of fluctuations in efficacy in Parkinson’s patients.	58.8%
*Topic 2: Role of COMT-Is in the treatment of fluctuations in effect*		
2.1	COMT-Is are only effective when used in combination with levodopa therapy.	84.7%
2.2	The efficacy of COMT-Is correlates with the levodopa dose.	45.5%
2.3	COMT-Is produce a more consistent dopaminergic effect.	90.9%
2.4	The effect of COMT-Is sets in early: immediately after the start of treatment.	45.9%
2.5	COMT-Is are effective treatments for both motor and non-motor fluctuations in effect.	93.7%
2.6	COMT-Is are the treatment of choice for managing fluctuations in efficacy.	58.2%
2.7	COMT-Is should be administered early in the treatment of fluctuating efficacy.	94.6%
2.8	COMT-Is can be safely combined with MAO inhibitors, including safinamide.	85.5%
*Topic 3: Comparative aspects of individual COMT-Is*		
3.1	All COMT-Is approved for the treatment of PD (opicapone, entacapone, tolcapone) are equally effective.	3.7%
3.2	Opicapone has the best safety profile of all COMT-Is.	54.1%
3.3	Opicapone is the easiest to manage in daily use due to its once-daily dosing and favourable safety profile.	82.6%
3.4	The switch from entacapone to opicapone can be made overnight.	83.6%
3.5	Possible side effects of opicapone are mainly determined by dopaminergic stimulation.	79.1%
3.6	Side effects such as diarrhoea or discolouration of the urine do not usually occur with opicapone.	83.6%
3.7	Adding COMT-Is to levodopa therapy is easy to manage due to opicapone’s once-daily dose.	95.5%
3.8	Tolcapone should only be considered as a reserve medication due to its side-effect profile.	83.6%

### Participants

The study was initiated by the German Delphi Panel, a group of six experts on PD from Germany, chaired by W.H. Jost, Wolfach/Homburg, and L. Tönges, Bochum. For the survey, the members of the editorial committee of the current German S2k guideline on PD, published by the German Society of Neurology (DGN), were initially contacted.

This group of experts was also able to provide further names of individuals who could be considered as potential additional participants. Physicians who were colleagues of the experts, who were already participating in the Delphi process, or who had any other potential conflict of interest were excluded. The final list of participants was sent to all of them so that any further individuals meeting the exclusion criteria could be excluded. However, this did not result in any further exclusions.

The 200 movement disorders experts thus identified were contacted by email and asked to participate. A total of 115 of those contacted gave their consent and took part in the survey. The authors would like to express their sincere thanks to these colleagues.

### Survey

An advisory board initially identified three key areas for the survey:

1. Variations in efficacy and the use of COMT-I.
2. The role of COMT-I in the treatment of fluctuations in efficacy.
3. Comparative aspects of individual COMT-I.

On this basis, the authors developed 22 statements (6 or 8 statements per topic area) for the survey through further consultation processes (see Table 1). The statements were made available to participants online via SurveyMonkey. Participants were asked to indicate, on a Likert scale of 1–5 (1: ‘strongly disagree’; 5: ‘strongly agree’), the extent to which they agreed with each statement. The analysis is based on responses of 4 (‘agree’) and 5 (‘strongly agree’) on the Likert scale.

## Results

### Topic area 1: variations in efficacy and the use of COMT-I

#### Ambiguous responses

**Table:** 

1.4	The assessment of motor and non-motor fluctuations requires systematic evaluations, such as the WOQ-9.	64%

The Wearing-off Questionnaire, 9-item version (WOQ-9) has become established in numerous clinics for the assessment of motor and non-motor fluctuations. The ambiguous response to this question likely indicates that the respondents’ experience with the questionnaire may have played a role. Participants who have routine experience of using the WOQ-9 may have answered this question in the affirmative. Participants who do not use such tests because they consider them too time-consuming or still too unreliable, on the other hand, have presumably given a negative response.

Yet the WOQ-9 could be an important diagnostic tool that, through appropriate adjustments to treatment, can contribute to a significant improvement in the quality of life of PD patients. For whilst patients report in the early years that the classic motor symptoms (bradykinesia, tremor and rigidity) are the most debilitating, they state later on – after six years – that when fluctuations occur, these are the most debilitating (Politis et al. 2010). 

In the DEEP study, the WOQ-19 (the original, comprehensive questionnaire for which the WOQ-9 was subsequently derived) demonstrated that medication fluctuations are underdiagnosed and can be diagnosed earlier than through clinical examination (Stocchi et al. 2014). 

Although a rate of 64% suggests general agreement, further studies would be required to substantiate this.

**Table:** 

1.6	COMT-Is may also be suitable for the treatment of on-off fluctuations in Parkinson patients.	58.8%

COMT-Is are approved as an add-on for the treatment of wearing-off and are very well suited (Höglinger et al. 2023, Product 2022, Product 2024, Product 2022). 

However, they are not approved for managing fluctuations in efficacy in the form of unpredictable OFF episodes. Because these episodes require a rapid onset of action, fast acting rescue therapies such as sublingual or subcutaneous apomorphine or inhaled levodopa are more appropriate for this purpose.

#### Affirmative responses

**Table:** 

1.1	Fluctuations in effect can manifest themselves in both motor and non-motor symptoms	99.1%
1.2	Motor fluctuations are diagnosed on the basis of the patient’s medical history and clinical examination.	93.0%
1.3	Non-motor fluctuations are diagnosed on the basis of the patient’s medical history and clinical/neuropsychiatric examination.	89.6%

PD is a progressive, neurodegenerative disorder characterised by the loss of dopaminergic neurons in the substantia nigra. The extensive loss of dopaminergic neurons reduces dopamine levels and leads to the onset of motor symptoms (Kalia and Lang 2015). However, motor dysfunction only occurs once dopamine levels have fallen by 70–80% (Werner and Coveñas 2014, Brunton 2018). Consequently, the diagnosis is often not made until this stage, as it is usually based on the cardinal symptoms.

For drug therapy in the early stages of the disease, the German S2K guideline recommends levodopa, MAO-B inhibitors (MAO-B-Is) and dopamine agonists (DAs), either as monotherapy or in combination (Höglinger et al. 2023). Due to the progressive loss of dopaminergic neurons in the substantia nigra, exogenously administered levodopa, or the resulting dopamine, can no longer be stored as the disease progresses. Consequently, the exogenously administered levodopa is converted into dopamine in the central nervous system and released in a pulsatile manner (Widnell 2005). According to the German S2K guideline, patients experience fluctuations in efficacy when, “with three daily doses of levodopa (approx. after 4–5 hours), there is a noticeable loss of efficacy before the next dose is taken” (Höglinger et al. 2023).

Statement 1.1 received the highest level of agreement in the entire survey. This indicates that there is broad acceptance among physicians in Germany that fluctuations in treatment response in PD can affect both motor and non-motor symptoms. There is also a very high level of consensus regarding statements 1.2 and 1.3. It is indisputable that motor fluctuations can be diagnosed through a clinical examination if one looks for them. Non-motor fluctuations can also be clearly diagnosed through a clinical/neuropsychiatric examination. In doing so, one should also proactively look for non-motor fluctuations. This is because they may occur before motor fluctuations (Poewe et al. 2017) and severely impair the quality of life of those affected. According to the DEEP study, the diagnosis of non-motor fluctuations is still frequently made too late (Stocchi et al. 2014). Both motor and non-motor fluctuations can be identified using the WOQ-19 and also the WOQ-9 (Kakimoto et al. 2023). 

**Table:** 

1.5	COMT-Is are suitable for the treatment of motor and non-motor fluctuations in Parkinson’s patients.	97.4%

Support for statement 1.5 is also very high. COMT-Is can block peripheral pathways of levodopa degradation, which significantly increases its bioavailability (Rocha et al. 2014). In doing so, COMT-Is not only raise levodopa levels, but also smooth them out and prolong the half-life of levodopa. A pharmacokinetic study demonstrated that opicapone, in particular, raises trough levodopa levels (Ferreira et al. 2022). It is precisely this effect that is required for the symptomatic treatment of PD: the associated increase in bioavailability and prolongation of the effect of levodopa keep patients within the therapeutic window for longer, which reduces their motor and dopa-responsive non-motor fluctuations and, consequently, improves their quality of life (Ferreira et al. 2022, Ferreira et al. 2016, Reichmann et al. 2022). 

### Topic 2: role of COMT-I in the treatment of fluctuations

#### Ambiguous findings

**Table:** 

2.2	The efficacy of COMT-Is correlates with the levodopa dose.	45.5%

The pharmacological mechanism of COMT-I is not dependent on the absolute levodopa dose, and clinical data show relevant benefits even at relatively low doses (≤ 400 mg). At higher levodopa doses, however, the risk of dyskinesias increases and levodopa dose adjustments may be required (Lee et al. 2024, Ferreira et al. 2024). 

In contrast, at higher levodopa doses, the risk of dyskinesias increases when a COMT-I is added, meaning that the levodopa dose must then be reduced. Ultimately, however, the aim of COMT inhibition is not to reduce the levodopa dose, but to smooth out levodopa levels – this is independent of the levodopa dose.

**Table:** 

2.4	COMT-Is take effect early on: immediately after treatment begins.	45.9%

Although the effect of COMT-Is begins on the first day, full efficacy is only achieved after a delay. In a pharmacokinetic study, opicapone was found to be superior to placebo as early as the first day in terms of maximum inhibition of COMT activity (E_max_) and inhibition of the extent of COMT activity (measured using the area under the effect-time curve, AUEC). However, these differences were significantly more pronounced after eight days (Rocha et al. 2013). A post-hoc analysis of the Phase III BIPARK I and II studies supported this: according to the results, the efficacy of opicapone was significant after one week, but reached its maximum within two to three weeks of starting treatment, subsequently plateaued and then remained stable at that level (Lees et al. 2016). 

**Table:** 

2.6	COMT-Is are the treatments of choice for managing fluctuations in efficacy.	58.2%

As agreement with statement 2.6 was only around 58%, COMT-Is do not appear to be regarded by physicians in Germany as the treatment of choice for fluctuations in efficacy. However, the question was presumably understood differently, as there was very high agreement (just under 95%) with statement 2.7, which states that COMT-Is should be administered early in the treatment of fluctuations in efficacy (see below).

Although these two answers may seem contradictory, we will return to this in Question 2.7.

#### Affirmative responses

**Table:** 

2.7	COMT-Is should be administered at an early stage in the treatment of fluctuations in efficacy.	94.6%

The high level of agreement with statement 2.7 appears, at first glance, to contradict the ambiguous response to statement 2.6, which states that COMT-Is are the treatment of choice for fluctuations in efficacy (see above).

Statement 2.6 can also be interpreted as ‘first choice’. Statement 2.7, on the other hand, is more broadly worded, as early use does not necessarily imply first-line treatment. Traditionally, COMT-I have not been regarded as the ‘first choice’ for the treatment of treatment fluctuations, with other classes of drugs being considered more favourable in this context. This now appears to be changing with opicapone, as a majority of 58% already regard COMT-Is as the treatment of choice for fluctuations in efficacy. And, as described later, the best choice for early intervention depends on several factors, such as patient preference.

These findings suggest that earlier use of COMT-I is beneficial (statement 2.7). As DDCI already provides single enzymatic inhibition and shifts levodopa metabolism towards the COMT pathway, the addition of COMT-I as a double enzymatic inhibition step is a logical next step. The optimization of levodopa metabolism through COMT-I is increasingly recognized as important, in contrast to the traditional use of COMT-I as a late-stage option in the treatment of off-phases.

Accordingly, just under 95% of participants suggest that many clinicians regard COMT-Is as a key early option for dopa-responsive fluctuations.

The current German S2k guideline lists the following options for the treatment of fluctuations: (Höglinger et al. 2023)

- Fractionation of levodopa doses and, if necessary, dose adjustment.
- Additional doses of levodopa preparations with modified formulations (soluble, inhaled or sustained-release levodopa).
- Additional doses of DAs.
- Additional doses of MAO-B-Is.
- Additional doses of COMT-Is.

It is added for context: (Höglinger et al. 2023)

- It is not possible to prioritize the individual therapeutic options for Parkinson’s patients with fluctuations based on studies.
- The individual sequence of treatment options should take into account the spectrum of action, the side-effect profile and the patient’s preferences.

Regarding the available COMT-Is, the German S2k guideline notes that, due to safety considerations, opicapone and entacapone should be used as first-line treatments and tolcapone as a second-line treatment under close safety monitoring (clinical and laboratory) (Höglinger et al. 2023). 

From the authors’ perspective, given its good efficacy, generally good tolerability and ease of use, opicapone is the preferred choice for COMT-I, to be initiated immediately following the diagnosis of motor fluctuations. Once treatment with opicapone is established, it is often sufficient over the long term to adjust only the levodopa dose as the condition progresses.

**Table:** 

2.5	COMT-Is are effective treatments for both motor and non-motor fluctuations in effect.	93.7%

The efficacy of COMT-Is in patients with wearing-off was demonstrated in the respective registration studies (Regensburger et al. 2023). In a randomized, double-blind, parallel-group study, entacapone significantly increased the mean ON time by 1.4 h from the start of the study to weeks 8–24 (*p* < 0.01) and, concomitantly, significantly reduced the mean OFF time by 1.1 h vs. baseline (*p* < 0.001). The difference between entacapone and placebo in ON time during weeks 8–24 was 1.3 h (Rinne et al. 1998). 

In the Phase III BIPARK I study, opicapone reduced the mean daily OFF time by an average of -1.95 h within 14–15 weeks (Ferreira et al. 2016). The reduction compared with baseline was significantly greater than with placebo (-0.93 h; *p* = 0.0015) and was not inferior to entacapone (-1.61 h; *p* = 0.0051) (Ferreira et al. 2016). A significant superiority of opicapone over placebo was also observed in the change in mean daily ON time without disabling dyskinesias (*p* = 0.002) (Ferreira et al. 2016). 

The benefits of opicapone were also evident in the assessments by clinicians (CGI-C) and patients (PGI-C). Both the clinicians and the patients scores were significantly improved only for opicapone, but not entacapone (Ferreira et al. 2016). 

Regarding the efficacy of COMT-Is on non-motor symptom fluctuations, the German real-world OPTIPARK study, for example, showed that under opicapone therapy, within three months there was a decrease in the proportion of patients reporting each of the nine symptoms recorded in the WOQ-9. At the same time, the mean total score on the Non-Motor Symptom Scale (NMSS) improved from 43 at the start of the study to 34 after three months (Reichmann et al. 2022). With regard to long-term effects, a retrospective data analysis after one year of opicapone therapy showed no significant change in the NMSS score (*p* = 0.605) and thus a stabilization of non-motor symptoms. After one year of entacapone therapy, a deterioration in the NMSS score had occurred (*p* = 0.005) (Leta et al. 2023). 

These findings are consistently supported by further prospective studies: for instance, the OPEN-PD study demonstrated (Santos García et al. 2022)– in line with the results from OPTIPARK – a significant improvement in total NMSS scores with opicapone. In addition, the OASIS study documented significant improvements in sleep-related symptoms, as measured by the PDSS-2 (Ferreira et al. 2025). 

With regard to non-motor symptoms (NMS), it should be noted that some NMS, including depression, constipation, pain, urogenital problems and sleep disorders, may respond to dopaminergic therapy, whereas other NMS are not dopa-responsive and therefore require different therapeutic strategies (Chaudhuri et al. 2006). 

**Table:** 

2.1	COMT-Is are only effective in combination with levodopa therapy.	84.7%

Levodopa has a short half-life and is metabolized, among other things, by COMT, resulting in the formation of 3-OMD, a pharmacologically inactive metabolite that cannot be converted into dopamine.

COMT-Is block this peripheral degradation pathway, thereby increasing the bioavailability of levodopa and ensuring that more of the substance is available for passage into the central nervous system and for conversion to dopamine. This established mechanism of action means that COMT-Is are effective only in combination with levodopa. Against this background, consistent agreement with such statements appears pharmacologically plausible.

**Table:** 

2.3	COMT-Is produce a more consistent dopaminergic effect.	90.9%

In a pharmacokinetic study, the addition of opicapone or entacapone resulted in a significant increase in levodopa trough levels (C_min_) compared with placebo (Rocha et al. 2014). Systemic levodopa exposure (measured by the area under the concentration-time curve, AUC) increased significantly with opicapone compared to placebo; no significant difference was observed when comparing entacapone with placebo. At the same time, opicapone and entacapone significantly inhibited maximum COMT activity (E_max_) and the extent of COMT activity (measured by the area under the effect curve, AUEC) compared with placebo, with the effect of opicapone on AUEC being significantly greater than that of entacapone (Rocha et al. 2014). 

It was also demonstrated for opicapone that, in addition to increasing levodopa trough levels, a smoothing of levodopa plasma concentrations over time is achieved. This is associated with more stable dopaminergic stimulation and is clinically reflected in a significant prolongation of ON time and a reduction in OFF time (Ferreira et al. 2022). 

**Table:** 

2.8	COMT-Is can be safely combined with MAO-B-Is, including safinamide.	85.5%

In theory, there is no evidence to suggest potential interactions between COMT-Is and MAO-B-Is. In the BIPARK registration trials for opicapone, the additional use of selegiline at a dose of up to 10 mg daily orally or 1.25 mg daily buccally, and of rasagiline at a dose of up to 1 mg daily, was permitted (Ferreira et al. 2016, Lees et al. 2017). A corresponding combination of opicapone with one of these MAO-B-Is was administered in the BIPARK-I study to approximately 20% of participants (Ferreira et al. 2016). At the time of opicapone’s authorization, there was no experience regarding combination with safinamide. According to the opicapone product information, concomitant use of rasagiline or selegiline is permitted, whereas caution is advised with safinamide (Product 2022). 

However, in the open-label Phase IV OPTIPARK study on the use of opicapone in routine clinical practice in Germany, 136 participants were co-medicated with rasagiline and 67 with safinamide (Reichmann et al. 2022). These subgroups were described separately (Pavese et al. 2021), but the treatment-related adverse events from the OPTIPARK study and the BIPARK studies were consistent. This provides initial evidence that the combination of opicapone and safinamide is also safe under real-world conditions.

In addition to several studies, a wealth of practical experience now also suggests that COMT-Is and MAO-B-Is can be combined without any problems. This is also reflected in the high approval rate of 85.5%.

### Topic 3: comparative aspects of individual COMT-I

#### Negative responses

**Table:** 

3.1	All COMT-Is approved for the treatment of PD (opicapone, entacapone, tolcapone) are equally effective.	3.7%

When approving new active substances, regulatory authorities require that they demonstrate the same or even greater efficacy than those previously used. This has a significant influence on the study designs for the development of new drugs and may lead to potential differences – whether positive or negative – being overlooked. It is therefore essential to continue to pay attention to such differences after approval.

Statement 3.1 may sound trivial at first glance, as the regulatory trials did indeed show comparable effects. However, the question of appropriate evaluation automatically leads to considerable tension here. The statement refers to the efficacy, not to the side effects, nor to differences in administration. When the first two COMT-Is were on the market, the superiority of tolcapone over entacapone was consistently claimed. However, this claim was soon met with numerous contradictions, as experienced specialists in movement disorders observed significant differences in many patients. When opicapone was introduced, the available data generally confirmed comparable efficacy with entacapone, with differences being merely suspected or actually observed only in isolated cases.

However, the assessment of this statement clearly contradicts this: only 3.7% of participants rate all three COMT-Is as equally effective; conversely, 96.3% see significant differences in the clinical efficacy of the three COMT-Is. Of course, it is relevant to ask where exactly these differences lie. In any case, almost all respondents assume that differences in the degree of efficacy exist.

This is also confirmed by relevant publications (Wu et al. 2024). A direct comparison of the efficacy of opicapone and entacapone in the treatment of motor ‘OFF’ phases, for example, demonstrated the superiority of opicapone and revealed differences between these two COMT-Is (Ferreira et al. 2016). There is no direct comparison of these two COMT-Is with tolcapone. Experts from the Movement Disorder Society agree that entacapone and opicapone cannot be considered equally effective (Bie et al. 2025). After evaluating the efficacy of COMT-Is in the treatment of motor fluctuations, they concluded that opicapone is ‘efficacious’ and entacapone is ‘likely efficacious’, where the differences in classification are mostly driven by quality and design of the available trials (Pavese et al. 2021). 

#### Ambiguous answers

**Table:** 

3.2	Opicapone has the best safety profile of all COMT-Is.	54.1%

It is unclear why there is such low agreement with this statement. The German S2k guideline classifies opicapone as the best-tolerated COMT-Is. According to the German S2k guideline, opicapone is also better tolerated than DAs (Höglinger et al. 2023). 

After years of experience with opicapone, the authors have not observed any relevant side effects attributable to opicapone itself. In contrast, entacapone and tolcapone may be associated with side effects that are (substance-specific e.g. diarrhea and discoloration of the urine with entacapone and tolcapone, and liver toxicity with tolcapone) (Product 2022, Product 2024). 

If dyskinesias occur while taking opicapone, these are not side effects but a consequence of the action of opicapone. In a one-year open-label extension of the BIPARK-I study, the incidence of dyskinesias increased significantly in patients who had received entacapone during the double-blind phase after they were switched to opicapone. This was controlled by adjusting, in particular reducing, the levodopa dose (Ceballos-Baumann et al. 2018), which is in line with established clinical strategies for the management of opicapone therapy.

In a post-hoc analysis of the BIPARK-I and -II registration studies, the incidence of dyskinesias was lower when patients received opicapone within the first year of the onset of motor fluctuations (Ebersbach et al. 2020). This is because, at this early stage, there are still sufficient dopaminergic cells present to temporarily store excess levodopa, thereby reducing the risk of dyskinesias (Lees et al. 2019). 

This is supported by the aforementioned ADOPTION study: (Ferreira et al. 2024) In the open-label Phase IV study, in which 92% of patients were receiving only three doses of levodopa per day when they started add-on therapy with opicapone, only 8% of participants developed dyskinesias.

Against this background, opicapone should be initiated as early as possible within the approved indication, and close attention should be paid to the possible occurrence of dyskinesias after the start of therapy, so that these can be controlled promptly, if necessary, by adjusting the levodopa regimen.

#### Affirmative responses

**Table:** 

3.5	Possible side effects of opicapone are mainly determined by dopaminergic stimulation.	79.1%

It has already been discussed in statement 3.2 that this statement is correct. The dual blockade of levodopa’s metabolic pathways through the combined administration of a DDC inhibitor such as carbidopa and the COMT-I opicapone effectively increases levodopa plasma levels, resulting in an increased risk of dyskinesias. As previously explained, peak levodopa levels—and thus the risk of dyskinesia—can be reduced if add-on therapy with opicapone is initiated early after the onset of the first fluctuations, whilst the levodopa storage function of the dopaminergic cells in the substantia nigra is still well preserved (Ferreira et al. 2024, Bie et al. 2025). In the later stages of the disease, dyskinesias may be become more increase. These correlations may explain the high level of agreement with statement 3.5. The remaining variability in the assessment can be understood as reflecting different clinical perspectives or interpretations.

**Table:** 

3.6.	Side effects such as diarrhea or discoloration of the urine do not usually occur with opicapone.	83.6%

Here too, the statement is correct and is therefore frequently agreed with. Nevertheless, around 20% of respondents disagreed. There are isolated reports of diarrhea occurring in connection with opicapone, but benserazide can cause diarrhea and can be the cause. In that case, switching from benserazide to carbidopa would resolve the problem (Ong et al. 2021). The prescribing information for opicapone does not list diarrhea or discoloration of the urine as side effects (Product 2022). 

**Table:** 

3.3	Opicapone is the easiest to manage in daily use due to its once-daily dosing and favorable safety profile.	82.6%
3.7.	The addition of COMT-Is to levodopa therapy is easy to implement due to opicapone’s once-daily dose.	95.5%

These two statements overlap considerably, so it makes sense to discuss them jointly. As previously mentioned, the German S2k guideline considers opicapone to be the best-tolerated COMT-I (Höglinger et al. 2023). The once-daily dosage increases convenience for patients and promotes adherence. When opicapone was first introduced, it was initially considered a drawback that, according to the product information, the COMT-I was to be taken once daily at bedtime, at least one hour before or after levodopa combination preparations, as this meant an additional time to take the medication.

In clinical practice, opicapone is increasingly taken simultaneously with the last evening dose of levodopa, preferably without food. This deviates from the current European SmPC (which specifies intake at least one hour before or after levodopa, based on the regimen used in the phase 3 pivotal trials). Supporting evidence remains limited: the food interaction is documented only in a conference abstract (Santos et al. 2017), and the only study addressing simultaneous dosing was a small, non-randomised, four-day observation with cross-over design (Huber et al. 2019) that found no difference in Unified Parkinson’s Disease Rating Scale (UPDRS) Parts III/IV outcomes. Prospective data from adequately powered studies are needed before this practice can be formally recommended.

In addition to once-daily dosing, add-on therapy with opicapone can even maintain the lower number of dosing times of levodopa, which can also contribute to good convenience and adherence (Ferreira et al. 2022, Ferreira et al. 2024). In the ADOPTION study, for example, in patients with motor fluctuations who were predominantly taking only three doses of levodopa per day, add-on therapy with 50 mg of opicapone per day showed greater efficacy than taking an additional 100 mg of levodopa per day (Ferreira et al. 2024). 

**Table:** 

3.4	The switch from entacapone to opicapone can be made overnight.	83.6%

Switching from entacapone to opicapone is very straightforward in everyday practice and is carried out routinely: on the last day of entacapone treatment, all entacapone doses are taken as usual, with the final entacapone dose in the evening being omitted and replaced by a dose of opicapone. From the following day, patients take opicapone once daily (Ferreira et al. 2017). Patients who have switched from entacapone to opicapone reported improvements in motor fluctuations (Ferreira et al. 2018).

Against this background, it is surprising that around 15% of respondents do not agree that switching COMT inhibition from entacapone to opicapone is possible overnight.

**Table:** 

3.8	Due to its side-effect profile, tolcapone should only be considered as a temporary medication or as a reserve treatment	83.6%

According to the prescribing information, tolcapone is used in combination with levodopa plus benserazide or carbidopa in patients with PD who respond to levodopa and experience fluctuations in motor function, but who do not respond to or cannot tolerate other COMT-Is. However, due to the risk of potentially fatal acute liver damage, tolcapone should not be considered as a first-line adjunctive therapy to levodopa/benserazide or levodopa/carbidopa (Product 2022). 

The German S2k guideline also states that, due to its hepatotoxicity, tolcapone should only be used as a second-line treatment and under close safety monitoring (clinical and laboratory) (Höglinger et al. 2023). 

It is therefore somewhat unclear why 16% of respondents disagree with this statement. This may be due to the wording ‘provisional medication’: patients who are on tolcapone and tolerate it well can continue to use it and do not need to switch.

## Discussion and conclusions

With 115 participating Parkinson’s experts from Germany, the survey provides significant insights into the current status of COMT-Is in the treatment of PD. As the responses indicate, there is consensus on the importance of motor and non-motor fluctuations and their anamnestic and clinical diagnosis. Opinions differ, however, regarding the use of the WOQ-9 for the systematic assessment of fluctuations, which has clear advantages. It is important to determine what the reasons for this might be: is the questionnaire, for example, insufficiently well-known, difficult to obtain, or too time-consuming to use? In daily practice, the time required is likely to be the decisive factor.

Only 58.2% of respondents agreed with the statement “COMT-Is are the treatments of choice for managing fluctuations in efficacy”. Whilst this is a narrow majority, it does not constitute clear agreement. In contrast, 95% considered the early use of COMT-Is for fluctuations in efficacy to be appropriate, which equates to unanimous agreement. The possible reasons for this apparent contradiction have already been discussed.

Apart from this, the survey on the role of COMT-Is in the treatment of fluctuations clearly shows that these:

- Should be used in combination with levodopa, and that this combination enables a more consistent dopaminergic effect.
- Are effective in both motor and non-motor fluctuations.
- Should be introduced at an early stage.
- Can be easily combined with other treatments, such as MAO-B-Is, including safinamide.

When comparing the three representatives of this class of substances, there is a consensus that:

- Not all COMT-Is are equally effective.
- Opicapone is the easiest to manage due to its safety profile and once-daily dosing.
- Patients already on levodopa therapy can be switched from entacapone to opicapone very easily.
- The switch from entacapone to opicapone is straightforward and can be done overnight.
- Possible side effects with opicapone are mainly attributable to its dopaminergic effect.
- Neither diarrhea nor discoloration of urine occurs with opicapone.
- Tolcapone – as described in the German S2k guideline and the product information – should only be considered as a reserve treatment.

To date, only symptomatic, dopaminergic therapy is available for PD (in the early and middle stages). Levodopa is the gold standard for this. If fluctuations in efficacy occur during this therapy, optimization of the treatment aims to raise the trough levels of levodopa so that patients remain within the therapeutic window for as long as possible and do not fall into an ‘OFF’ phase. At the same time, the peak levels of levodopa should, as far as possible, not rise so high as to cause dyskinesias.

The role of COMT-Is, which increase the bioavailability of levodopa and smooth out its therapeutic levels, is clearly defined in this context. Their positive effect on many motor and non-motor symptoms has also been clearly demonstrated. According to the results of pharmacokinetic studies, COMT inhibition with opicapone in particular can significantly increase the troughs of levodopa levels without significantly raising the peaks, which corresponds exactly to the objectives of treating fluctuations in levodopa levels in PD. According to the German S2k guideline, opicapone is also considered the best-tolerated COMT-I.

There were also minor discrepancies between the Q&A and the current guideline. This is partly due to the fact that the guideline focuses on published data, whereas clinical experience also plays a major role in the Q&A. For example, if the marketing authorization for opicapone states that it should be taken one hour before the last dose of levodopa, this is the current position. However, if in clinical practice the two doses are now frequently taken together, this is standard practice and deviates from both the marketing authorization and the guidelines. Accordingly, the marketing authorization and the guidelines could be amended in future.

Taking into account the characteristics of the three COMT isoforms and the available study results, the authors believe that opicapone should be the treatment of choice and should be initiated as soon as possible following the diagnosis of fluctuations – perhaps even as the first add-on therapy to levodopa.

## Data Availability

No datasets were generated or analysed during the current study.
